# Supplementary material for: Evaluation of Triclosan coated suture in obstetrical surgery: A prospective randomized controlled study (NCT05330650)
Source: PLoS One. 2022 Dec 15;17(12):e0278939. doi: 10.1371/journal.pone.0278939 (PMC9754295; doi:10.1371/journal.pone.0278939)
Supplement: S1 Dataset — (DOCX) [file pone.0278939.s001.docx]

[10.6084/m9.figshare.21330624](https://doi.org/10.6084/m9.figshare.21330624)
