# Supplementary material for: Evaluation of Triclosan coated suture in obstetrical surgery: A prospective randomized controlled study (NCT05330650)
Source: PLoS One. 2022 Dec 15;17(12):e0278939. doi: 10.1371/journal.pone.0278939 (PMC9754295; doi:10.1371/journal.pone.0278939)
Supplement: S2 File — (DOC) [file pone.0278939.s003.doc]

**INFORMED CONSENT FORM**

*I, the undersigned ........................................................., agree to participate in the research project entitled Evaluation of Triclosan Coated Suture in Obstetrical Surgery: A Prospective Randomized Controlled Study*

*I have read the information letter concerning the above-mentioned research project.*

*I acknowledge that I have received clear and understandable information about the project and that I have received answers to all my questions about the project.*

*I have had sufficient time to make a decision.*

I have been informed, orally and in writing, of the objectives of the project, its data collection methods and the terms of my participation in the project by Dr. ........................

I have also been informed:

How the investigators will keep my information confidential

My right, as a voluntary participant in this study, to withdraw at any time if I deem it necessary without any prejudice to me.

My right to contact, if I have any questions about the project, the Principal Investigator of the project: Dr. Hajer Bettaieb ; Telephone : +21697696936; Email : hajer.bettaieb@fmt.utm.tn

Consequently: I freely and voluntarily accept that my personal data related to the study may be accessible to the study managers and possibly to the health authorities. With the exception of these persons, who will process the information in the strictest respect of medical secrecy, my anonymity will be preserved.

Done at ..............., on .....................Consent collected by ……………...

Participant's name .................................. and signature ........................
