# Supplementary material for: Evaluation of Triclosan coated suture in obstetrical surgery: A prospective randomized controlled study (NCT05330650)
Source: PLoS One. 2022 Dec 15;17(12):e0278939. doi: 10.1371/journal.pone.0278939 (PMC9754295; doi:10.1371/journal.pone.0278939)
Supplement: S3 File — (DOCX) [file pone.0278939.s004.docx]

**Evaluation of Triclosan suture in obstetrical surgery**

**Study protocol of a randomized controlled single center trial**

**Introduction :**

Surgical site infection (SSI) presents a burden to healthcare with significant morbidity and mortality(1).

SSI increase direct and indirect costs with a significant overall financial burden for health care system(2). In fact, prolonged hospital stays, increasing rate of readmission and reintervention, are associated with SSI(3).

Several risk factors of SSI had been reported, one of these factors is microorganism colonization of suture materials(4). To prevent this risk, triclosan-coated polyglactin sutures were developed. Triclosan is a phenolic antiseptic with a broad spectrum of antibacterial and antifungal properties and low toxicity. In fact, the concentration used to impregnate or suture poses no risk to humans or the environment. There are no reported carcinogenic, mutagenic or teratogenic effects with such concetration(1). Triclosan gained US food and drug administration approval in 2002 to coat suture materials(5).

Effectiveness of Triclosan coated suture in reducing SSIs was demonstrated in several studies and metanalyses(5–7). Despite these broad evidences, the use of Triclosan coated suture has not been evaluated in obstetric surgery especially in countries with high SSIs incidence.

The main of our study is to determinate whether the use of Triclosan coated suture (Vicryl Plus®) influences the rate of SSI after caesarian delivery (CD) compared to similar Vicryl. Secondary objectives are to analyze contributing risk factors and evaluate the cost impact of antimicrobial suture use.

**Hypotheses**

- The use of antibacterial skin sutures reduces the rate of SSI after ceasarian delivery.

-Triclosan coated suture reduce healthcare cost.

**Methodes :**

**Study designe :**

This is a mono-centric prospective controlled randomized trial conducted in the departement of obstetrics and gynecology of Ben Arous hospital-Tunisia.

It is designed as a triple blind parallel-group superiority trial.

Patients candidate to caesarian section will be informed about the study. Informed consent will be signed by each participating patient and a surgeon of the department before the surgery.

**Data collection and management :**

For data collection we will use an anomymos canevas (Appendix 1), containing six sections: SSI risk factors / Admission / Operative procedure / follow up / SSI diagnosis and management / hospital cost.

Data were collected from: Prenatal follow-up records/ Obstetrical records /Surgical reports/Anesthesia reports/Post-operative monitoring records.

The confidentiality of the participants will be maintained at any time.

The following clinical characteristics will be collected : Age, smoking, Body mass index, presence of diabetes, presence of anemia and history of CD.

Risk factors related to the current pregnancy, will be collected : the presence of gestational diabetes (GDM), preeclampsia (PE) as well as the delay of premature rupture of membranes (PROM) if it exists.

The following information will be collected at the time of the operation : the nature of the caesarean section, operating time, operative complication, the Amerian Society of Anasthesiologist (ASA) score, and the use of an unloading drainage system.

During the postoperative visits, signs of surgical site infections will be cheked , the healing time and the quality of the scar at 30 days will also be noted.

In the presence of a SSI we note its nature, in-patient or out-patient management, antibiotic therapy used, local care kits, imaging examination requested, radiological drainage if needed and the frequency of investigation tests performed.

**Outcomes :**

*Primary outcome:* The rate of SSIs within 30 days after surgery.

*Secondary outcomes*: The rate of wound healing complications.

The cost impact of triclosan coated suture use from a hospital perspective.

***Inclusion criteria:***

Will be include in the study, pregnant woman with elective or emergency caesarian delivery.

***Non-inclusion criteria***:

Suspected or confirmed chorioamniotitis \ SARS-COV2 infection. Patients without signed informed consent were not included.

***Exclusion criteria:***

Cases with incomplete follow-up will be exclude from the study.

***Sample size calculation:***

The rate of SSI after Caesar section in our center was 9% in 2019. Thus, the assumed expected wound infection rates were 2% with coated suture use versus 9% for the control group. On the basis of a two-sided chi2 test for equality of proportions, the study was expected to have 80% power to detect a relative risk reduction of 5%. A total of 296 patients were estimated to be needed, that is 148 in each arm. The G*Power 3.1.9.6 software (Heinrich-Heine University, Dusseldorf, Germany) was used to calculate the sample size.

***Randomization:***

After delivering a written consent, patients candidate to caesarian section will be assigned randomly by a computer generated list, to closure with polyglactin non coated suture VICRYL (Groupe 1), or with polyglactin coated suture VICRYL Plus (Groupe 2). The randomization will be performed by Random Allocation Software 1.0.0 (Freeware) by M.Saghaei, MD.

The suture materials for each group will be placed in an opaque sealed box (Box 1 for group 1) and (Box 2 for group 2). The only one allowed to fill the boxes is the senior surgeon responsible of this study

***Surgical procedure:***

A perioperative antibiotic prophylaxis will be administered to all patients.

Per operative hair removal will be prescribed.

Pfannenstiel incision will be performed in all cases.

Uterine and aponeurosis closure will be performed with an overlock suture with 1/0 thread.

Gloves will be changed after aponeurosis closure.

Subcutaneous tissue will be sutured with 2/0 thread.

Skin will be closed with intradermic suture with 3\0 thread.

In the operative room, a scrub nurse verifies the randomization list and delivers the suture material crimped after removing the package. Macroscopically it is impossible to distinguish Vicryl from Vicryl plus.

***Diagnosis of SSI:***

We adopted the Centers for Disease Control definition (CDC) to diagnose SSI(8):

Superficial SSI Infection is associated with skin and subcutaneous tissue within 30 days after surgery and at least one of the following:

Purulent drainage with or without culture confirmation

Positive tissue or fluid culture

Deep SSI Infection associated with deep tissues within 30 days after surgery and at least one of the following:

Purulent drainage from the deep incision.

A deep incision spontaneously dehisces or opened by the surgeon with at least one of fever, pain, tenderness—unless culture is negative

Radiologically or histologically detected abscess or deep infection

Diagnosis of deep incisional infection made by a surgeon or attending physician

***Follow up:***

Patients will be followed 30 days after surgery. Four visits were programmed after surgery :

**Check-up 1:** Two days after surgery.

**Check-up 2:** 7 days after surgery.

**Check-up 3:** 15 days after surgery.

**Check-up 4:** 30 days after surgery.

In each visit, wound surveillance will be performed by a resident, and if an SSI is suspected the wound will be photographed and the patient will be referred to a senior surgeon to confirm the diagnosis (figure2).


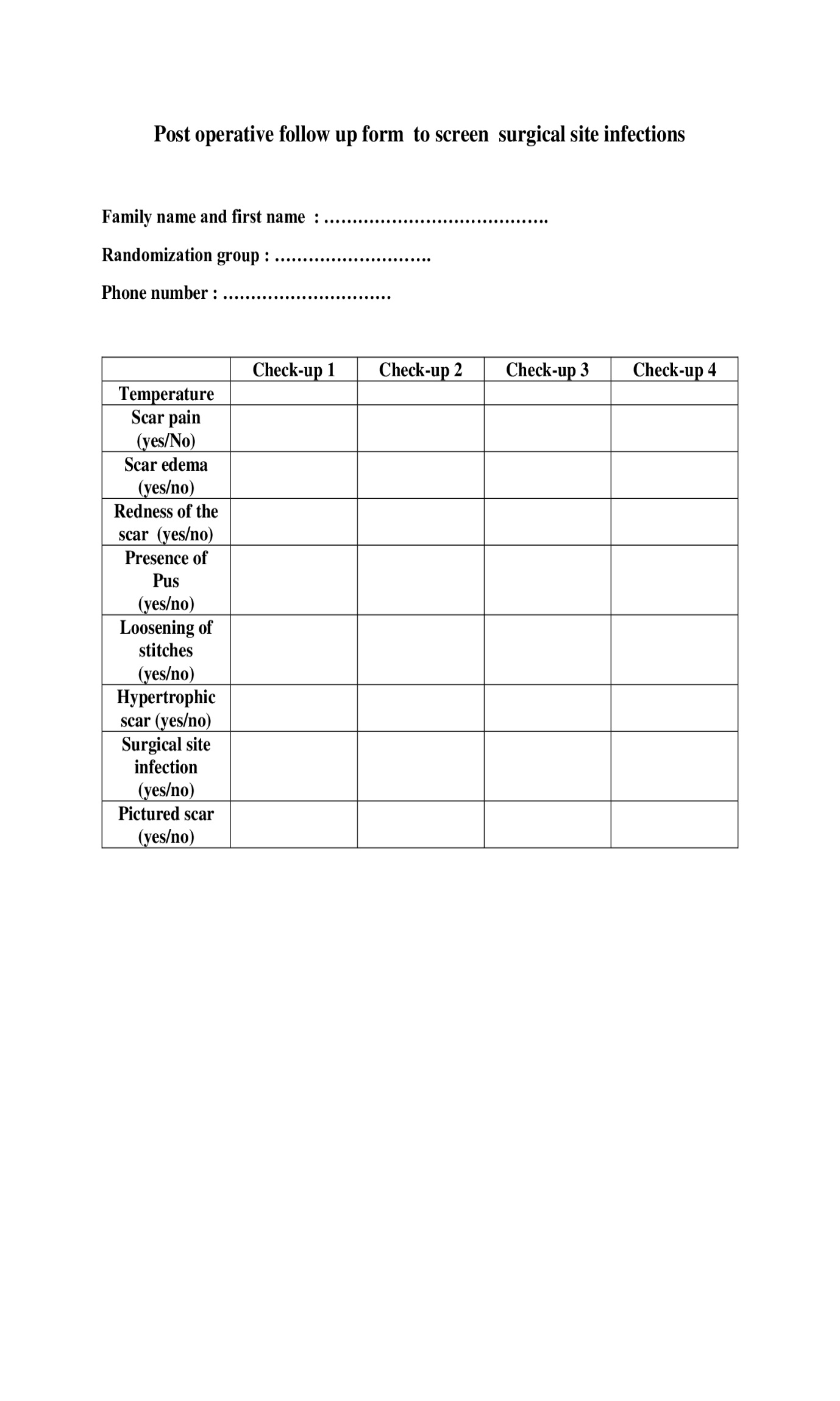


***Costs estimation:***

**Cesarian cost:** In all cases we will use a surgical bundle with fixed cost. The overall cost per procedure is calculated by the following formula:

**Cesarian cost = Bundle cost + Suture material cost + Hospital stay cost**.

**SSI cost:** A care bundle with fixed cost will be also used for wound care. The overall cost per SSI is calculated by the following formula:

**SSI cost = SSI bundle cost + Drugs cost + Investigation tests cost + Reintervention cost + Hospital stay cost.**

**Total cost (TC)**: The total cost is the sum of cesarian cost and SSI cost

**Total cost = Cesarian cost + SSI cost**

**Cost per SSI prevented = TC (Vicryl Plus) – TC (Vicryl) / SSI (Vicryl Plus) – SSI (Vicryl).**

**Limitations:**

The fact that the survey was carried out in a single public institution without extending to other health structures, constitutes one of the limitations of our study. Another limitation is that data were not computerized.

**Ethical Considerations :**

The participating women will be informed of the study protocol and will sign a written consent form .

Data will be collected anonymously.

Photographs of the scars will be taken after the women had been constantly observed, respecting modesty and anonymity and eliminating any details that might identify the patient.

We declare on our honor not to have any real or potential conflict of interest with the theme of the study. The antibacterial thread used is the only one available in Tunisia. The threads supply are provided by the hospital pharmacy.

**References:**

1. Leaper D, Assadian O, Hubner NO, McBain A, Barbolt T, Rothenburger S, et al. Antimicrobial sutures and prevention of surgical site infection: assessment of the safety of the antiseptic triclosan. Int Wound J. 2011 Dec;8(6):556–66.

2. Mattavelli I, Rebora P, Doglietto G, Dionigi P, Dominioni L, Luperto M, et al. Multi-Center Randomized Controlled Trial on the Effect of Triclosan-Coated Sutures on Surgical Site Infection after Colorectal Surgery. Surg Infect. 2015 Jun;16(3):226–35.

3. Bustamante Montalvo M, Cainzos M, Prieto Carreiras L, Castiñeira Piñeiro A, García Iglesias A, Fernandez Novo A, et al. Evaluation of the effect of triclosan coated sutures in the prevention of surgical site infections in a Spanish hospital setting: A prospective, observational study. Infect Prev Pract. 2021 Sep 1;3(3):100154.

4. Sandini M, Mattavelli I, Nespoli L, Uggeri F, Gianotti L. Systematic review and meta-analysis of sutures coated with triclosan for the prevention of surgical site infection after elective colorectal surgery according to the PRISMA statement. Medicine (Baltimore). 2016 Aug;95(35):e4057.

5. Ahmed I, Boulton AJ, Rizvi S, Carlos W, Dickenson E, Smith NA, et al. The use of triclosan-coated sutures to prevent surgical site infections: a systematic review and meta-analysis of the literature. BMJ Open. 2019 Sep 3;9(9):e029727.

6. Nakamura T, Kashimura N, Noji T, Suzuki O, Ambo Y, Nakamura F, et al. Triclosan-coated sutures reduce the incidence of wound infections and the costs after colorectal surgery: a randomized controlled trial. Surgery. 2013 Apr;153(4):576–83.

7. Arslan NC, Atasoy G, Altintas T, Terzi C. Effect of triclosan-coated sutures on surgical site infections in pilonidal disease: prospective randomized study. Int J Colorectal Dis. 2018 Oct;33(10):1445–52.

8. Guideline for Prevention of Surgical Site Infection, 1999. Centers for Disease Control and Prevention (CDC) Hospital Infection Control Practices Advisory Committee - PubMed

**Appendix**

**
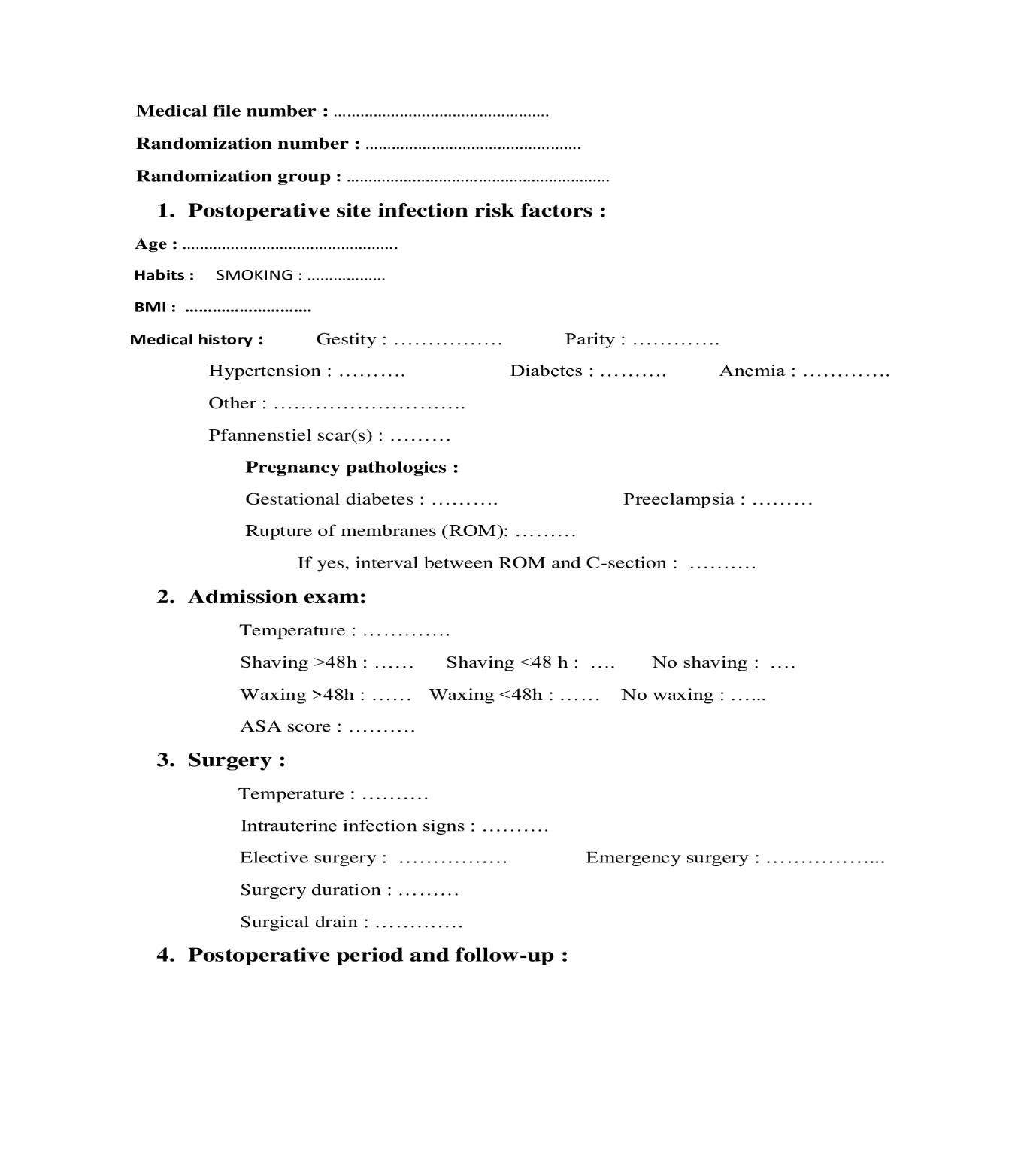
**

**
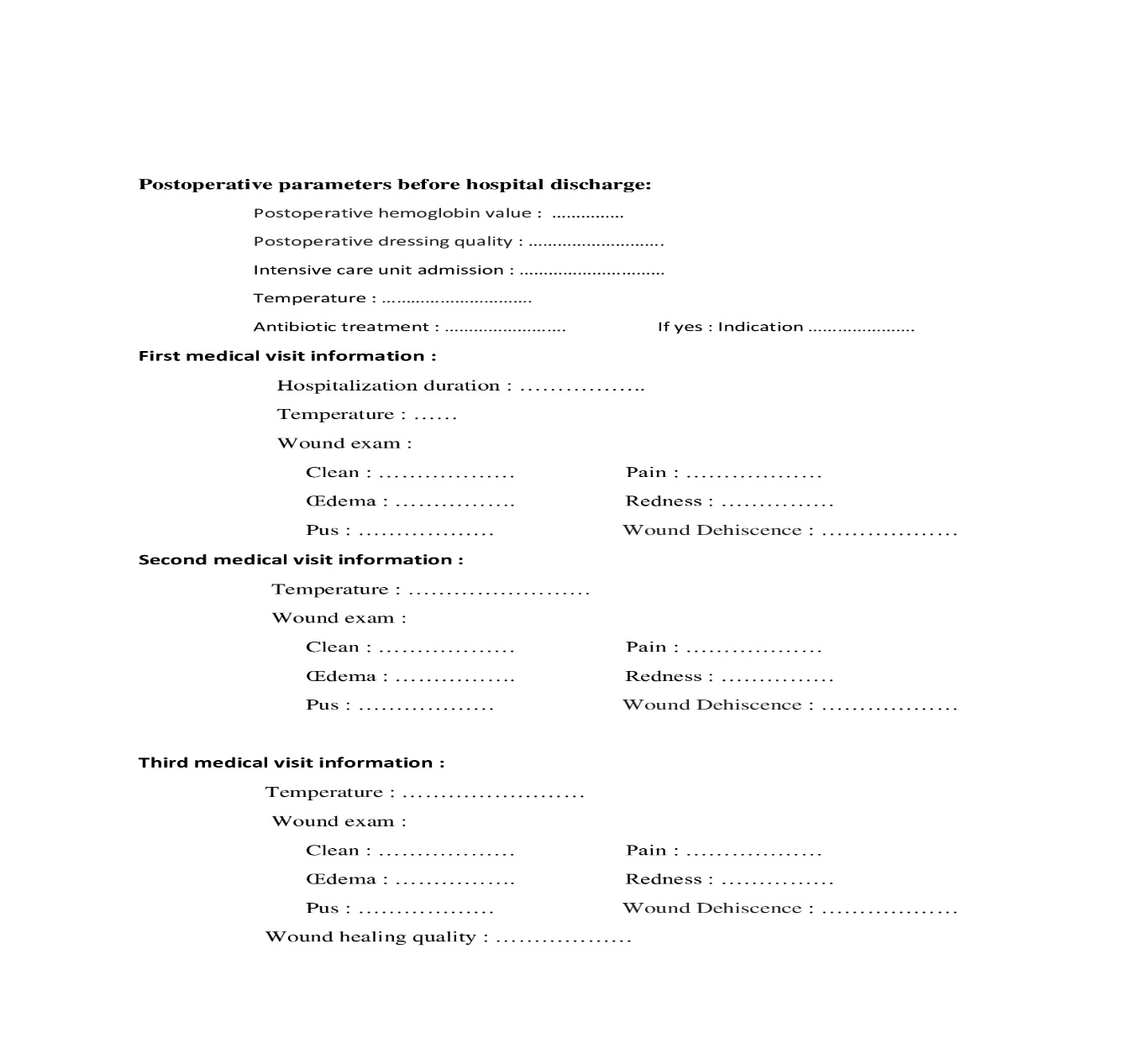
**

**
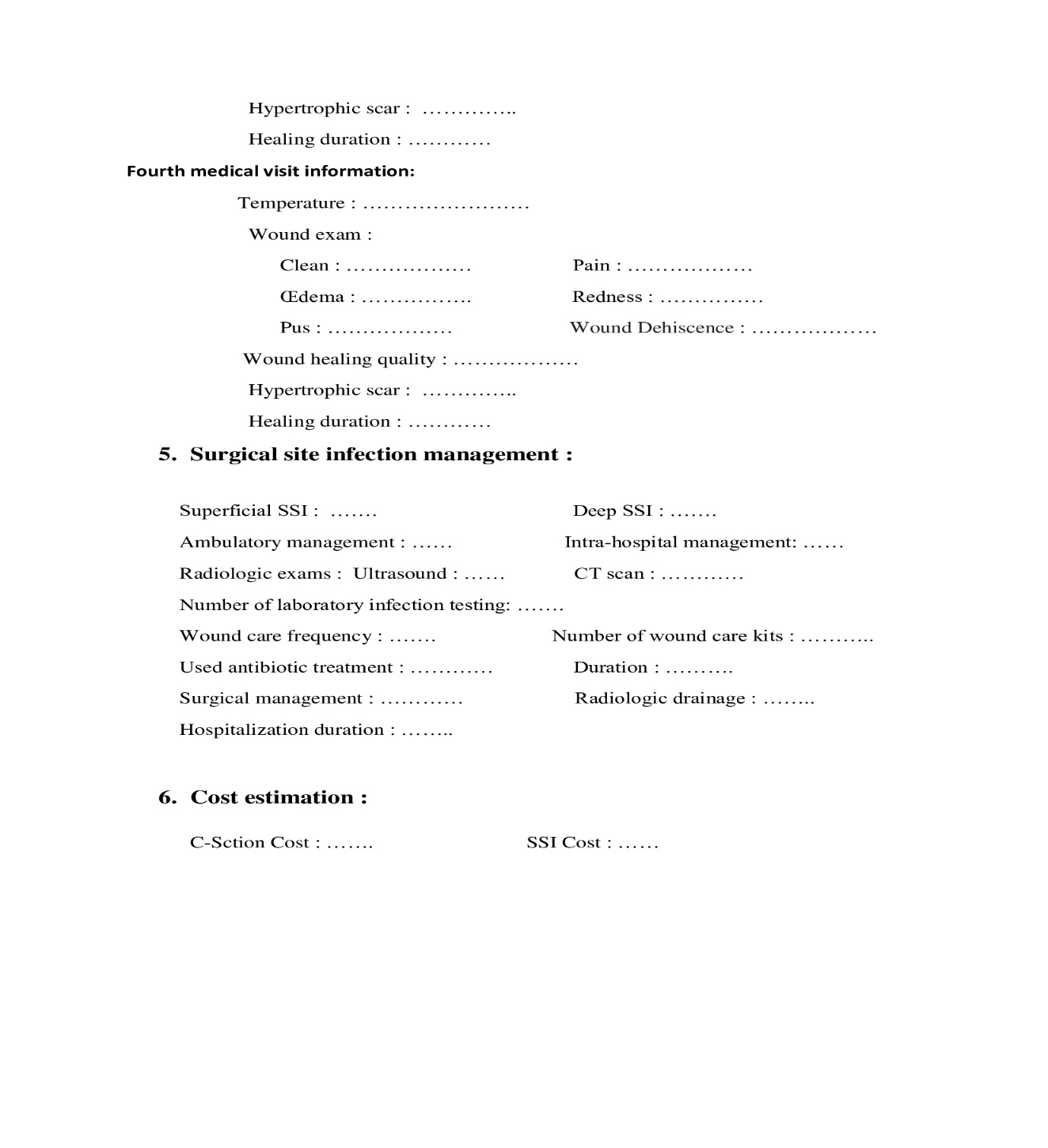
**

**Appendix 1 : Data collection form**
