# Supplementary material for: Evaluation of Triclosan coated suture in obstetrical surgery: A prospective randomized controlled study (NCT05330650)
Source: PLoS One. 2022 Dec 15;17(12):e0278939. doi: 10.1371/journal.pone.0278939 (PMC9754295; doi:10.1371/journal.pone.0278939)
Supplement: Attachment — Submitted filename: RESPONSE to REVIEWERS.docx [file pone.0278939.s005.docx]

Manuscript [PONE-D-22-20609]

Response to Reviewers

**Response to Reviewers**

Thank you for giving us the opportunity to submit a revised draft of the manuscript “Evaluation of Triclosan Coated Suture in Obstetrical Surgery: A Prospective Randomized Controlled Study”. We appreciate the time and the effort that you and the reviewers dedicated to provide feedback on our manuscript and we are grateful for the insightful comments and valuable improvements to our paper. We have incorporated most of the suggestions made by the reviewers. Those changes are highlighted within the manuscript. Please see below for a point-by-point response to the reviewers’ comments and concerns. All page numbers refer to the revised manuscript file with tracked changes.

**Reviewers' Comments to the Authors:**

**Reviewer 1:** This manuscript tried to assess the effectiveness of Triclosan coated sutures in reducing SSI, the rate of wound healing complications, and the cost impact of triclosan-coated suture use from a hospital perspective. I have reviewed the manuscript and would like to suggest following changes in a section-wise manner. Overall, there are many grammatical and typographical mistakes.

Comment from reviewer one concerning the Abstract: Abstract seems well written.

Author response: Thank you.

Comment from reviewer 1 concerning specific change in the introduction: This section needs to be well written and arranged into relevant paragraphs. The authors should first talk in brief about the SSI, risk factors, basics of the suture in the discussion, and how sutures are related to the development of SSI. Following this, the authors should mention recent studies pertaining to the topic and highlight the literature gap. Then, the authors should state how the current study aims to fill the gap.

Author response: We agree with the reviewer’s assessment. Accordingly, we have revised the introduction. We included SSI risk factors in page 2 (line 50 to 54), basics of suture and relation between suture and SSI in page 2 (line 55 to 60). Recent studies were cited in page 2 (line 60 to 65). We tried to explain gap in literature in page 3 (line 66 to 70).

Comment from reviewer 1 concerning the methodology: The authors have mentioned inclusion, non-inclusion and exclusion criteria. However, there should be only inclusion and exclusion criteria. There are grammatical and typographical mistakes. Such as chi2 should be replaced with chi-square, Caesar section with caesarean section, and many more. Also, format the section in proper paragraphs based on the journal’s guidelines. Please make sure to double-check and address all the mistakes.

Author response: Thank you for pointing this out. Section format was modified. We removed the non-inclusion criteria and we tried to correct all grammatical mistakes by a double-check (page 4 to page 8).

Comment from reviewer 1 concerning the section “Results”: Please format the tables based on the journal’s guidelines. The statistical values mentioned in brackets are confusing.

Author response: Thank you for pointing this out. All tables format has been modified based on journal’s guidelines.

Comment from reviewer 1 concerning the section “Discussion”: The first paragraph should mention the noteworthy findings of the study. The authors should compare and contrast these findings with other studies in subsequent paragraphs. The discussion is too brief. Also, author should add a paragraph regarding limitations and future implications of this study.

Author response : We tried to mention our finding in the first paragraph of the discussion in page 13 (line 239 to 243) and finding were compared as recommended in page 14 (line 253 to 260). A paragraph that mention the limitation of the study were added in page 15 (line 286 to 293).

Comment from reviewer 1 concerning the section “Conclusion”: The conclusion is well written.

Author response: thank you.

Comment from reviewer 1 concerning the section “References”: Please format the references based on the journal’s guidelines.

Author response : section references has been modified based on journal’s guidelines.

**Reviewer 2:** A mono-centric prospective controlled randomized trial in pregnant women with elective or emergency caesarian delivery. Patients, surgeons and outcome’s assessor triple blinded to treatment. Three hundred eighty patients were randomly assigned to closure with polyglactin non coated suture VICRYL, or with polyglactin coated suture VICRYL Plus after caesarian section. The primary outcome was the rate of SSIs within 30 days after surgery. SSI rate was 2.5% in Triclosan group compared to 8.1% with non-coated

suture. Use of Triclosan coated suture was associated with 69% reduction in SSI rate (p = 0.037; OR:0.294; CI95%:0.094 – 0.921). The authors claim a total of 5312 USD (33 USD per procedure) was saved with TCS coated suturing; 590 USD per prevented SSI.

Comment from reviewer 2 concerning the trial registration: No trial registration given.

It seems to be this trial: https://ichgcp.net/clinical-trials-registry/NCT02847936

However, the sample size in that registered trial is 400 and not 296 patients.

Author response: Thank you for pointing this out. The trial registration was not mentioned in the manuscript. Our trial registration does not correspond to the trial mentioned by the reviewer.

The trial registration figures in page 1 at the title and the section “Ethics statement” at page 4 (line 72) in the revised manuscript.

Comment from the reviewer 2 concerning “Sample size calculations”: expected wound infection rates were 2% with coated suture use versus 9% for the control group. On the basis of a two-sided chi2 test for equality of proportions, 80% power to detect a relative risk reduction of 5%, 296 patients were needed, 148 patients per arm. The sample size is powered to test a null hypothesis of this difference: 9% vs 2% is an ARR of 7% and a RRR of 78%. The actual results showed an ARR of 5.6% (8.1-2.5%) and a RRR of 69%. This means that the null hypothesis may be false as the study is underpowered to correctly test a smaller difference than 7%. In other words, the conclusion of the effectiveness of triclosan coated sutures needs to be toned down considerably, coming from an underpowered study.”

Author response: We agree that this is a potential limitation of the study and we tried to figure this weakness and to explain it in the section “Discussion” at the page 15 (line 288 to 293).

Comment from the reviewer 2 concerning “Skin preparation: What was used as standard skin prep? Specify at 'surgical procedure'.

Author response: The skin preparation used was added in section “Surgical procedure’ at page 6 (Line 113).

Comment from the reviewer 2 concerning “The randomization procedure”: “a scrub nurse verified the randomization list..." (p. 12) Explain this. How could there be a list that the scrub nurse was allowed to look into. Proper computer randomization allocates treatment group per patient separately. A pre-randomized list of treatment allocations does not count as proper randomization.

Author response: We agree that the section randomization lacks details and was not well described. In the revised manuscript we tried to clarify and to meticulously describe the procedure of randomization in the section “Randomization and blinding” at the page 5 (line 95 to 104).

Comment from the reviewer 2 concerning “the wound surveillance”: who did the wound surveillance and decides whether or not a patient was seen by a senior surgeon for suspicion of wound infection?

Author response: Thank you for pointing this out. Wound surveillance was made by the resident in charge on the ward. Page 6 (line 124).

Comment from the reviewer 2 concerning “Cost estimation”: Cost estimation section needs to be deleted completely, as well as the related conclusion. This is not a proper nor adequate economic analysis. 'Cesarian cost = Bundle cost + Suture material cost + Hospital stay cost' and 'SSI cost = SSI bundle cost + Drugs cost + Investigation tests cost + Reintervention cost + Hospital stay cost' is not accurate, oversimplified, no details supplied, and only a very rough 'quick & dirty' estimate which must be left out because of imprecision and not following the rules of cost analyses.

Author response: We agree with the reviewer that the method to estimate cost was not appropriate and the section and its related conclusion were deleted from the revised manuscript.

Comment from the reviewer 2 concerning the section “Statistical analysis”: Statistical analyses paragraph is too concise, lacks detail.

Author response: We tried to clarify the paragraph by listing and describing all the statistical methods used for the analysis at page 7 and 8 (line 154 to 167).

Comment from the reviewer 2 concerning Table 1: Table 1 baseline characteristics. Since this is a RCT, no statistics must be performed on baseline characteristics as randomization should take care of random distribution. Delete p value column and related text in results.

Author response: We totally agree with the reviewer and p value column and related text was deleted.

Comment from the reviewer 2 concerning Table 2: Table 2 risk factor for SSI does not contribute to trial results presentation.

Author response : While we appreciate the reviewer’s feedback, we respectfully disagree. Since development of SSI is multifactorial, the bivariate analysis was conducted to define confounding factors.

Comment from the reviewer 2 concerning “Multivariate analysis”: p.19 'In multivariate regression, only use of non-coated suture was associated with a higher risk of SSI.' But none of the multivariate data are shown. Do we simply need to believe the authors without any possibility of peer review of the multivariate data? It's also not made available in supplementary materials.

Author response: As suggested results of multivariate analysis were added in the revised manuscript at page 12.

**Reviewer 3:** This randomized controlled trial assessed the effectiveness of Triclosan coated suture in reducing SSIs rate after caesarian delivery, and to evaluate the cost impact of antimicrobial suture use. They had interesting finding. And the study is well written. All information about the procedure was described in detail in Method section. Only one minor concern 'CI95%' should be '95%CI'.

Author response: Thank you.

**Reviewer 3:** A randomized controlled study was conducted which aimed to assess the effectiveness of Triclosan coated suture in reducing surgical site infections (SSIs) after cesarean delivery. The secondary aim was to compare rates of wound healing complications. A statistically significant reduction in the SSI rate was observed with the Triclosan coated suture compared to the control. Furthermore, Triclosan was associated with statistically lower risks of wound oedema, dehiscence and hematoma compared to the control.

Comment from the reviewer 3 concerning the section “Statistical analysis”:

Provide a comprehensive “Statistical analysis” section. List and describe all the statistical methods used for the analysis.

Page 4, last line: Write out chi-square

The statements “p degrees of significance” and “the results were presented with their p” are confusing. Please clarify or restate.

Indicate the type of nonparametric tests used and the specific data that was analyzed with these tests..

Author response: We tried to clarify the paragraph by Listing and describing all the statistical methods used for the analysis at page 7 and 8 (line 154 to 167).

Comment from the reviewer 3 concerning the section”Abstact”: Define the abbreviation SSI.

Author response : Thank you for pointing this out. The abbreviation has been defined.

Comment from the reviewer 3 concerning the section “Statistical analysis”:

Page 4, last line: Write out chi-square

The statements “p degrees of significance” and “the results were presented with their p” are confusing. Please clarify or restate.

Indicate the type of nonparametric tests used and the specific data that was analyzed with these tests.

Comment from the reviewer 3 concerning “Providing percentage”: Provide corresponding percentages for the counts in these sentences.

A. “One hundred seventy women were allocated in group 1 and 170 in group 2.”

B. “Ultimately, 160 patients remained in the Triclosan arm and 158 in the control arm

Author response: Thank you for pointing this out. Percentage has been added at page 8 (line 169 to 174).

Comment from the reviewer 3 concerning: Define groups 1 and 2 at first mention.

Author response: Thank you for pointing this out. The two groups were defined as recommended at page 5 (Line 93 and 94).

Comment from the reviewer 3 concerning” Table 1”: In addition to means, provide standard deviations. In addition to counts, provide percentages. If the data is not normally distributed, provide median, first and third quartiles.

Author response: Standard deviation, percentage were added in table 1 to normally distributed data and median, first and third quartiles were also mentioned. Page 9

Comment from the reviewer 3 concerning “Adding standard deviation”: Page 11, Second paragraph: Provide 95% confidence intervals for the 2.5% and 8.1%.

Author response: These two percentages define the rate of SSI in the two groups. No standard deviation could be calculated.

Comment from the reviewer 3 concerning “Multivariate analysis”:

In the “Statistical analysis” section indicate that both univariate and multivariate analysis were performed. The univariate results are shown in Table II. Clarify.

Clarify which factors remained in the multivariate regression.

Author response: As suggested results of multivariate analysis were added in the revised manuscript at page 12.

Comment from the reviewer 3 concerning: In the “Statistical analysis” section list and thoroughly describe the use of all statistical methods. Indicate if univariate or multivariate logistic regression was used for the wound healing disturbances (results shown on page 13 and Table III). For further clarity, in the title of Table III, indicate if the results are univariate or multivariate.

Author response: Thank you for pointing this out. In the revised manuscript we had mentioned that univariate analysis was used to compare wound healing disturbances at page 8 line 165.

Comment from the reviewer 3 concerning: Providing the discharge rates and summarizing the healing times in the two groups.

Author response: We described these two parameters at page 12 (line 224 to 226) and in Table 3.

Comment from the reviewer 3 concerning “Hospital cost”: Provide measures of dispersion for each hospital cost. Indicate if the summary statistics are means, medians, etc.

Author response: The section of hospital cost has been deleted as recommended.

Comment from the reviewer 3 concerning “Adding line numbering to the document” : To assist in the review process, add line numbering to the document.

Author response: Thank you it was a very helpful suggestion.

**Reviewer 4**: A randomized controlled study was conducted which aimed to assess the effectiveness of Triclosan coated suture in reducing surgical site infections (SSIs) after cesarean delivery. The secondary aim was to compare rates of wound healing complications. A statistically significant reduction in the SSI rate was observed with the Triclosan coated suture compared to the control. Furthermore, Triclosan was associated with statistically lower risks of wound oedema, dehiscence and hematoma compared to the control.

Major revision
Provide a comprehensive “Statistical analysis” section. List and describe all the statistical methods used for the analysis.

Author response: the statistical analysis section was revised. We tried to clarify the paragraph by listing and describing all the statistical methods used for the analysis at page 7 and 8 (line 154 to 167).

Minor revisions:
1- Abstract: Define the abbreviation SSI.
2- Page 4, last line: Write out chi-square.
3- Page 5, Randomization: Provide further details of the randomization process. If block randomization was used, state the block size.
4- Page 8, Statistical analysis: The statements “p degrees of significance” and “the results were presented with their p” are confusing. Please clarify or restate.
5- Page 8, Statistical analysis: Indicate the type of nonparametric tests used and the specific data that was analyzed with these tests.
6- Page 8: Provide corresponding percentages for the counts in these sentences.
A. “One hundred seventy women were allocated in group 1 and 170 in group 2.”
B. “Ultimately, 160 patients remained in the Triclosan arm and 158 in the control arm . . .”
7-Page 8: Define groups 1 and 2 at first mention.
8- Table I: In addition to means, provide standard deviations. In addition to counts, provide percentages. If the data is not normally distributed, provide median, first and third quartiles.
9- Page 11, Second paragraph: Provide 95% confidence intervals for the 2.5% and 8.1%.
10- In the “Statistical analysis” section indicate that both univariate and multivariate analysis were performed. The univariate results are shown in Table II. Clarify.
11- Page 13, lines 1-3: Clarify which factors remained in the multivariate regression model.
12- In the “Statistical analysis” section list and thoroughly describe the use of all statistical methods. Indicate if univariate or multivariate logistic regression was used for the wound healing disturbances (results shown on page 13 and Table III). For further clarity, in the title of Table III, indicate if the results are univariate or multivariate.
13- Page 13: Provide the discharge rates and summarize the healing times in the two groups.
14- Page 14: Provide measures of dispersion for each hospital cost. Indicate if the summary statistics are means, medians, etc.
15- To assist in the review process, add line numbering to the document.

Thank you for appointing this out. All minor revision were considered and clarified as recommended by reviewer n°4.
