# Supplementary material for: Evaluation of Triclosan coated suture in obstetrical surgery: A prospective randomized controlled study (NCT05330650)
Source: PLoS One. 2022 Dec 15;17(12):e0278939. doi: 10.1371/journal.pone.0278939 (PMC9754295; doi:10.1371/journal.pone.0278939)
Supplement: Attachment — Submitted filename: RESPONSE 2[14639].docx [file pone.0278939.s006.docx]

Manuscript [PONE-D-22-20609]

Response to Reviewers

Response to Reviewers

Thank you for giving us the opportunity to submit a revised draft of the manuscript “Evaluation of Triclosan Coated Suture in Obstetrical Surgery: A Prospective Randomized Controlled Study”. We appreciate the time and effort that you and the reviewers dedicated to provide feedback on our manuscript and we are grateful for the insightful comments on and valuable improvements to our paper. We have incorporated most of the suggestions made by the reviewers. Those changes are highlighted within the manuscript. Please see below, in blue, for a point-by-point response to the reviewers’ comments and concerns. All page numbers refer to the revised manuscript file with tracked changes.

**Reviewers' Comments to the Authors:**

**Reviewer 1 :**  Dear Author, I have reviewed the manuscript and unfortunately, I will not be able to recommend it for publication. This manuscript needs to be extensively edited for english language. Discussion section is not written well.

Author response : We tried to write the manuscript in a clear language and to correct grammatical and typographical errors.

**Reviewer 3** : All my comments have been addressed. I think the paper is suitable for publication in PLOS ONE. Thanks again

Author response : Thank you.

**Reviewer 4:**

Comment from reviewer 4 concerning bivariate analysis “Lines 159 & 165: State the statistical method(s) used for the bivariate and univariate analyses. “

Author response: Thank you for pointing this out. We tried to describe all statistical methods used for the bivariate analysis in the page 7 and 8 (line 156 to line 159).

Comment from reviewer 4 concerning Table I, Table II and Table IV:

To improve clarity, place "n=160" in the Group 1 header and "n=158" in the Group 2 header. Remove these numbers from the cell entries.

Table II:

a- In the title state the statistical method used for the bivariate analysis.

b- Provide a measure of dispersion for all means.

c- In similar fashion to comment #2 above, include the sample sizes in the header row and remove them from the individual cells.

Table IV: In similar fashion to comment #2 above, include the sample sizes in the header row and remove them from the individual cells.

Author response: We modified Tables as recommended.

Comment from reviewer 4 concerning Providing 95% confidence intervals for the proportions “Lines 190 and 195: Provide 95% confidence intervals for the proportions: 5.3%, 2.5%, and 8.1%. In the Statistical Analysis section, include the statistical method used to estimate the Cis.

Author response: Thank you for the reminder. 95%Cis were provided as recommended and method used was included in page 8 (line 165 to line 166).
